# Leaf nutrient resorption of two life-form tree species in urban gardens and their response to soil nutrient availability

**DOI:** 10.7717/peerj.15738

**Published:** 2023-07-19

**Authors:** Ruyuan Hu, Tairui Liu, Yunxiang Zhang, Rongrong Zheng, Jinping Guo

**Affiliations:** 1 College of Urban and Rural Construction, Shanxi Agricultural University, Taigu, Shanxi, China; 2 Shanxi Key Laboratory of Functional Oil Tree Cultivation and Utilization, Taigu, Shanxi, China; 3 College of Forestry, Shanxi Agricultural University, Taigu, Shanxi, China

**Keywords:** Garden tree species, Green and senesced leaves, Life form, Soil nutrient, Nutrient resorption, Nutrient limitation

## Abstract

**Background:**

Leaf nutrient resorption is a key strategy in plant conservation that minimizes nutrient loss and enhances productivity. However, the differences of the nutrient resorption among garden tree species in urban ecosystems were not clearly understood, especially the differences of nitrogen resorption efficiency (NRE) and phosphorous resorption efficiency (PRE) between evergreen and deciduous trees.

**Methods:**

We selected 40 most generally used garden tree specie belonged two life forms (evergreen and deciduous) and investigated the nitrogen (N) and phosphorus (P) concentrations in green and senesced leaves and soil nutrient concentrations of nine samples trees for each species. Then, the nutrient concentrations and resorption efficiency were compared, and the soil nutrients utilization strategies were further analyzed.

**Results:**

The results showed that the N concentration was significantly higher in the green and senesced leaves of deciduous trees than in the leaves of evergreen trees. The two life-form trees were both N limited and evergreen trees were more sensitive to N limitation. The NRE and PRE in the deciduous trees were significantly higher than those in the evergreen trees. The NRE was significantly positively correlated with the PRE in the deciduous trees. As the soil N and P concentrations increased, the nutrient resorption efficiency (NuRE) of the evergreen trees increased, but that of the deciduous trees decreased. Compared with the deciduous trees, the evergreen trees were more sensitive to the feedback of soil N and P concentrations. These findings reveal the N and P nutrient resorption mechanism of evergreen and deciduous trees and fill a gap in the understanding of nutrient resorption in urban ecosystems.

## Introduction

Nutrient availability is important for plant productivity (Chen & Chen, 2022). Nitrogen (N) and phosphorus (P) are the most common limiting elements for woody plant growth (Zhang et al., 2021), and their ratio can indicate the presence of a nutrient limitation (Hong et al., 2022; Zhang et al., 2022). Before shedding leaves, a plant tissue or organ transfers part of its nutrients (mostly N and P) to other living tissues or organs. This process is known as nutrient resorption (Lu et al., 2018). This technique, which is regarded as a crucial nutrient conservation mechanism (Wang et al., 2020), can increase nutrient use efficiency, increase the retention period of nutrients in plants and lessen their reliance on soil nutrients (Yang & Yang, 2021). Soil nutrient availability (Xie, Shan & Zhang, 2022) and stoichiometry (Tully et al., 2013) influence the nutrient resorption together. Studies have shown that NuRE decreases with increasing available soil nutrients (Wang et al., 2014). Nutrient transport between soil and plants heavily depends on the decomposition of leaf litter and the supply of soil nutrients (Li et al., 2022). In addition, the rate of nutrient loss in senesced leaves is thought to be a feedback as part of the dynamics of soil nutrition. The nutrient concentrations remaining in senesced leaves in turn affect their decomposition rates and soil nutrient availability (Tully et al., 2013).

There are some differences in nutrient resorption among different life forms (such as evergreen and deciduous, conifers and broadleaved) at different scales (Leon-Sanchez et al., 2020). At the global scale, nitrogen resorption efficiency (NRE) and phosphorous resorption efficiency (PRE) are lower in evergreen trees than in deciduous trees (Vergutz et al., 2012). However, NRE and PRE were shown to be higher in conifers than in broadleaved trees in a study of 137 woody species in six different forest types in northern China (Tang et al., 2013). Some scholars also believe that there are no differences in nutrient resorption between deciduous and evergreen trees (Aerts & Chapin, 2000). There are differences in soil nutrient absorption during leaf nutrient resorption in different life forms. The NRE and PRE in evergreen trees, which are usually dominant in barren environments (Yuan et al., 2018), are similar to or even lower than those in deciduous trees in nutrient-rich soils. Some researchers believe that plant functional types contributed more to differences in NRE than in PRE, while climate and soil have a greater impact on differences in PRE (Wang et al., 2020; Tang et al., 2013). However, the differences in urban garden tree species remain unclear.

Urban garden trees are an important part of urban green spaces (Hu et al., 2022) . According to the niche differentiation of garden trees, different life-form tree species are planted together to reduce the intense competition among species for the same nutrients and make full use of soil nutrients (Hu et al., 2022). This is the key to building a conservation-minded landscape and “ecological garden city” and improving the urban environment and ecosystem stability (Orti et al., 2022). It also serves as the basis for achieving a “carbon emission peak and carbon neutrality” (Feng et al., 2022) and is the only way to promote sustainable urban development (Wang et al., 2022). Due to increasing human disturbance, the soil nutrient dynamic balance in urban ecosystems has greatly changed or even been destroyed in recent years (Sifton et al., 2022), and the ecological strategies of plants, especially their nutrient strategies, have changed correspondingly. However, the strategies of different life-form tree species in urban ecosystems have not been thoroughly studied (Leon-Sanchez et al., 2020).

Studying the nutrient utilization strategies of different life-form tree species can contribute to understanding the relationship between nutrient utilization strategies among different life forms and soils and exploring the adaptability of different life forms and the nutrient cycle in urban ecosystems. By investigating the leaves and soil of 40 typical garden tree species in Taiyuan city and studying the relationship between green leaves, senesced leaves, and soil nutrient concentrations and N:P, we aim to describe the nutrient cycling mechanism from a stoichiometric perspective. We hypothesized the following:

1. The differences between the two life-form garden tree species result in N and P utilization strategies, mainly due to the differences in the nutrient concentrations and nutrient resorption efficiencies of evergreen and deciduous trees.

2. Evergreen and deciduous trees are limited by different nutrient types under the same urban landscape background.

3. The nutrient utilization strategies of the leaves of evergreen trees are more sensitive to soil nutrient concentration feedback than those of deciduous trees.

## Materials & Methods

### Study site

The research was performed in Taiyuan city, Shanxi Province, China, at 37.27°N and 38.25°N. Taiyuan is located in central Shanxi Province and is surrounded by mountains on three sides(west, north and east) and the Jinzhong Basin on one side (south). The overall topography is high in the north and low in the south, and the Fenhe River flows through the city. With an average annual temperature of 9.5 °C and 456 mm of precipitation, the city has a warm temperate continental monsoon climate. The zonal soil type is mainly brown soil. Site condition differences among urban areas are mainly reflected in the limited differences in groundwater levels and soil characteristics caused by distance to a river.

### Experimental design

Based on the total number and spacial frequency of the tree species (evergreen: deciduous = 1: 5.83) used in Taiyuan city, we selected 40 most generally used tree species belonging to two life forms of six evergreen trees and 34 deciduous trees.

**Figure 1:**
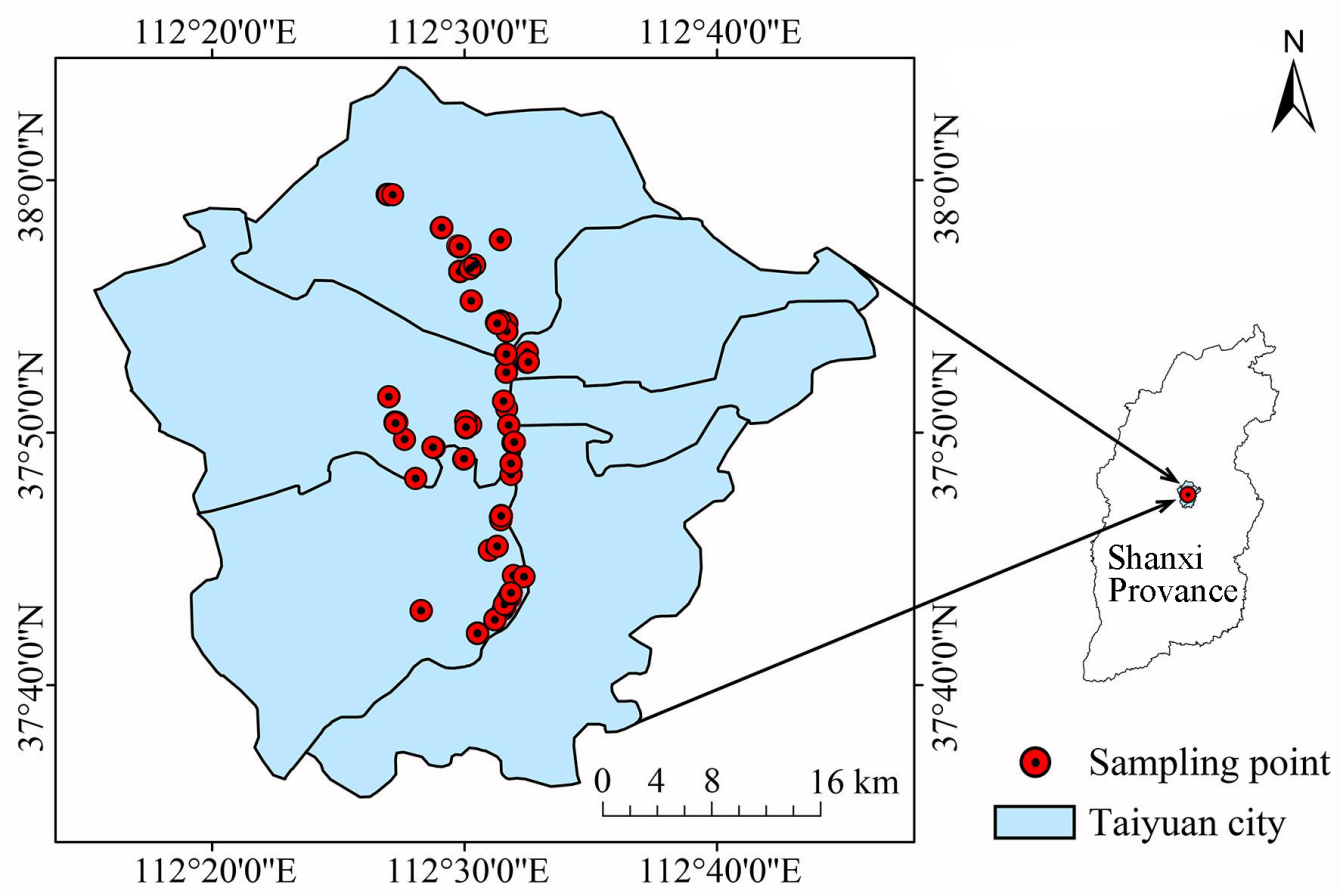
Lacation of tree sampling point.

To ensure representativeness and facilitate observation and sampling, the selected sample site was along the East Riverside Road. At the same time, the sample sites and management conditions were consistent. Among the three selected tree species, each tree species met the criteria of good growth, similar age, thorax/ground diameter, plant height and crown width. Each sample site contained three sample trees belonging to the same tree species. For each tree species, nine sample trees were selected from three sample sites. In total, 120 sample points (Fig. 1) and 360 sample trees were selected for 40 tree species (Table 1).

**Table 1:** Basic information of the sample tree species.

**Life form**	**Tree species**	**TH/m**	**DBH or DS/cm**	**CD/m**
Evergreen	*Pinus tabulaeformis*	7.10 ± 0.75	14.93 ± 2.66	1.87 ± 0.64
	*Pinus bungeana*	7.53 ± 1.08	13.93 ± 2.10	2.10 ± 0.62
	*Picea meyeri*	3.73 ± 0.64	6.93 ± 2.80	1.10 ± 0.35
	*Sabina chinensis*	7.57 ± 0.21	17.00 ± 2.17	1.27 ± 0.42
	*Sabina procumbens*	0.70 ± 0.42	4.48 ± 0.23	1.46 ± 0.39
	*Euonymus kiautschovicus*	1.30 ± 0.26	3.98 ± 0.14	0.94 ± 0.13
Deciduous	*Sophora japonica*	11.40 ± 0.26	15.57 ± 3.71	2.70 ± 0.70
	*Robinia pseudoacacia*	10.50 ± 1.10	23.50 ± 3.04	3.60 ± 0.82
	*Euonymus maackii*	2.87 ± 0.42	4.07 ± 0.12	1.13 ± 0.35
	*Populus tomentosa*	15.73 ± 1.10	32.67 ± 4.93	5.60 ± 0.53
	*Populus alba*	14.50 ± 0.14	29.50 ± 3.54	3.75 ± 0.35
	*Ginkgo biloba*	9.90 ± 1.37	14.63 ± 0.32	1.80 ± 0.44
	*Malus micromalus*	3.17 ± 0.15	7.12 ± 0.45	1.53 ± 0.39
	*Prunus cerasifera*	5.43 ± 0.60	7.10 ± 2.23	1.80 ± 0.44
	*Fraxinus chinensis*	6.97 ± 0.49	14.17 ± 2.84	2.07 ± 0.46
	*Salix babylonica*	10.43 ± 1.21	28.17 ± 1.26	4.30 ± 1.92
	*Salix matsudana*	10.23 ± 0.67	27.03 ± 4.36	3.73 ± 0.95
	*Albizia julibrissin*	7.85 ± 0.64	14.60 ± 0.85	2.90 ± 0.85
	*Acer negundo*	6.65 ± 0.49	21.70 ± 3.76	2.47 ± 0.50
	*Acer truncatum*	10.43 ± 0.93	21.90 ± 3.38	3.43 ± 1.03
	*Cotinus coggygria*	7.70 ± 0.26	5.87 ± 0.31	2.83 ± 0.35
	*Koelreuteria paniculata*	7.70 ± 0.61	13.90 ± 3.22	2.57 ± 1.03
	*Morus alba*	7.90 ± 0.14	10.47 ± 1.33	4.27 ± 1.80
	*Broussonetia papyrifera*	6.60 ± 0.28	8.70 ± 0.99	3.65 ± 3.32
	*Eucommia ulmoides*	9.37 ± 0.32	19.80 ± 3.60	2.73 ± 0.95
	*Rhus typhina*	6.47 ± 0.35	8.53 ± 0.93	2.67 ± 0.67
	*Quercus wutaishanica*	9.23 ± 2.80	17.40 ± 1.04	3.07 ± 0.61
	*Crataegus pinnatifida*	4.57 ± 0.40	14.50 ± 1.65	2.70 ± 0.36
	*Armeniaca sibirica*	5.47 ± 0.15	8.60 ± 0.28	2.57 ± 1.31
	*Amygdalus davidiana*	4.60 ± 0.50	7.25 ± 0.64	1.93 ± 0.32
	*Cerasus serrulata*	4.50 ± 0.50	13.00 ± 1.41	1.75 ± 0.35
	*Amygdalus triloba*	2.80 ± 0.35	3.90 ± 0.78	1.90 ± 0.20
	*Syringa oblata*	2.45 ± 0.07	3.23 ± 0.14	1.98 ± 0.46
	*Sophora japonica var. japonica f. pendula*	2.33 ± 0.25	11.73 ± 3.04	1.83 ± 0.40
	*Forsythia suspensa*	2.35 ± 0.35	4.12 ± 0.15	1.89 ± 0.54
	*Hibiscus syriacus*	2.70 ± 0.46	2.67 ± 0.61	1.17 ± 0.45
	*Sorbaria sorbifolia*	1.30 ± 0.28	3.37 ± 0.12	0.31 ± 0.11
	*Lonicera japonica*	3.15 ± 0.49	2.97 ± 0.16	1.15 ± 0.23
	*Cornus alba*	1.60 ± 0.36	3.45 ± 0.92	0.39 ± 0.14
	*Berberis thunbergii*	0.63 ± 0.15	3.25 ± 0.15	0.32 ± 0.12

**Notes.**

TH tree height DBH diameter at breast height (trees) DS diameter of seedling (shrubs) CD crown diameter

### Sampling and measurement

Green leaf and soil samples were collected during mid-July 2021. Senesced leaf samples were collected in November 2021. Green leaf samples were collected from the upper, middle, and lower parts of the crown to create a mixed sample. The 0–30 cm soil samples were taken by a soil drill after the litter removed firstly. For shrubs, soil samples were collected from three sites at 120° angles away from the crown radius. For trees, they were collected from three sites at 120° angles 0.5 m away from the trunk. Senesced leaf samples were collected from the leaves remained on the stalk but fell off naturally when the trees were gently shaken. The green leaf samples, the soil samples and the senesced leaf samples came from three sample trees in each sample site were respectively mixed to one composite sample. Totally, 120 green leaf samples, 120 senesced leaf samples and 120 soil samples were collected.

The leaf samples were first dried at 105 °C before being dried at 65 °C to a constant weight. The dried leaves and soil were then crushed and passed through a 100-mesh sieve. The total nitrogen (TN) and total phosphorus (TP) concentrations (Table S1) were measured with the colorimetric method. The process involved adding 5 ml H_2_SO_4_ and 6 ml H_2_O_2_ to the plants and mixing 1.85 g catalyst and 5 ml H_2_SO_4_ with the soil. Then, the samples were digested in a deboiling furnace, filtered at a constant volume, and finally analyzed by an automatic discontinuous chemical analyzer (Smartchem 450) (Hu et al., 2022). The soil concentrations of NH${}_{4}^{+}$-N, NO${}_{3}^{-}$-N, NO${}_{2}^{-}$-N and available P (AP) (Table S2) were also tested with the colorimetric method.

### Data analysis

Accounting for the leaf mass loss when leaves senesce, we recalculated the senesced leaf nutrient concentrations to compensate for the underestimation of NuRE using the mass loss correction factor (MLCF) (Van Heerwaarden, Toet & Aerts, 2003). The MLCF values varied according to life forms: 0.745 for conifers, 0.780 for evergreen broadleaved trees and 0.784 for deciduous broadleaved trees (Vergutz et al., 2012). In this research, all the data on nutrients in senesced leaves (Nu_sen_) underwent quality correction. The calculated Nu_sen_ was also considered the nutrient utilization efficiency (Tang et al., 2013). The NuRE was quantified as follows:

\begin{eqnarray*}\text{NuRE}= \frac{{\mathrm{Nu}}_{\mathrm{gr}}-{\mathrm{Nu}}_{\mathrm{sen}}}{{\mathrm{Nu}}_{\mathrm{gr}}} \times 100\text{%} \end{eqnarray*}

where Nu_gr_ represents the amount of nutrients in green leaves, while Nu_sen_ represents the amount of nutrients in senesced leaves.

The N in green leaves (N_gr_), P in green leaves (P_gr_), N in senesced leaves (N_sen_), P in senesced leaves (P_sen_), NRE, PRE and soil nutrient data were log10-transformed to meet the assumption of normality. One-way ANOVA was employed to analyse the N and P concentrations and N:P in green and senesced leaves between the two life forms. Additionally, the T test with Bonferroni adjustments was used to examine differences in the characteristics of nutrient absorption between the two life forms. A linear fitting equation was employed to analyse the correlations between soil parameters and the leaf index for the two life forms. Redundancy analysis (RDA) was used to identify the soil factors that explained the differences in N and P utilization between the two life-form tree species. We calculated the sorting axis lengths, and the lengths were all less than 3. We also calculated the variance inflation factor (VIF) for all soil factors using variance inflation factor analysis, and all VIF values were less than 10. All data are the mean ± SD. All data analyses were conducted in R 4.2.1 (R Core Team, 2022) and IBM SPSS Statistics 20 (SPSS, Inc., Chicago, IL, USA).

## Results

### N and P concentrations and N:P in green and senesced leaves between the two life forms

The N (*F* = 46.17, *p* = 0.000) and P (*F* = 29.94, *p* = 0.000) concentrations and N:P (*F* = 5.20, *p* = 0.023) were significantly higher in the green leaves than in the senesced leaves of the 40 typical garden tree species (Table 2). There were significant differences between the N (*F* = 10.219, *p* = 0.002) and P (*F* = 5.455, *p* = 0.021) concentrations and N:P (*F* = 12.611, *p* = 0.001) in green leaves between the two life forms (Table 2). Senesced leaves between the two life forms exhibited significant differences in N (*F* = 12.802, *p* = 0.001) concentrations and N:P (*F* = 10.595, *p* = 0.001) and nonsignificant differences in P concentration (Table 2). Additionally, except for the P concentration in the senesced leaves, the N and P concentrations and N:P in the evergreen trees were significantly lower than those in the deciduous trees (Table 2).

**Table 2:** N and P concentrations and N:P in green and senesced leaves of evergreen and deciduous trees.

**Leaf**	**Life form**	**n**	N (g kg^−1^)	P (g kg^−1^)	**N:P**
Green leaf	Evergreen	18	6.38 ± 1.60a	2.65 ± 0.43a	2.45 ± 0.69a
	Deciduous	102	14.61 ± 6.52b	2.94 ± 0.70b	5.04 ± 1.99b
	All	120	13.38 ± 6.72A	2.90 ± 0.68A	4.65 ± 2.07A
Senesced leaf	Evergreen	18	3.18 ± 0.44a	1.75 ± 0.36a	1.88 ± 0.47a
	Deciduous	102	5.93 ± 2.65b	1.61 ± 0.36a	3.78 ± 1.70b
	All	120	5.51 ± 2.64B	1.63 ± 0.37B	3.50 ± 1.72B

**Notes.**

n sampling point number

Different lowercase letters in the same column showed significant differences between the evergreen and deciduous trees, and different uppercase letters in the same column showed significant differences between the green and senesced leaves at *p* < 0.05.

### NRE, PRE and their correlated relationship between the two life forms

**Figure 2:**
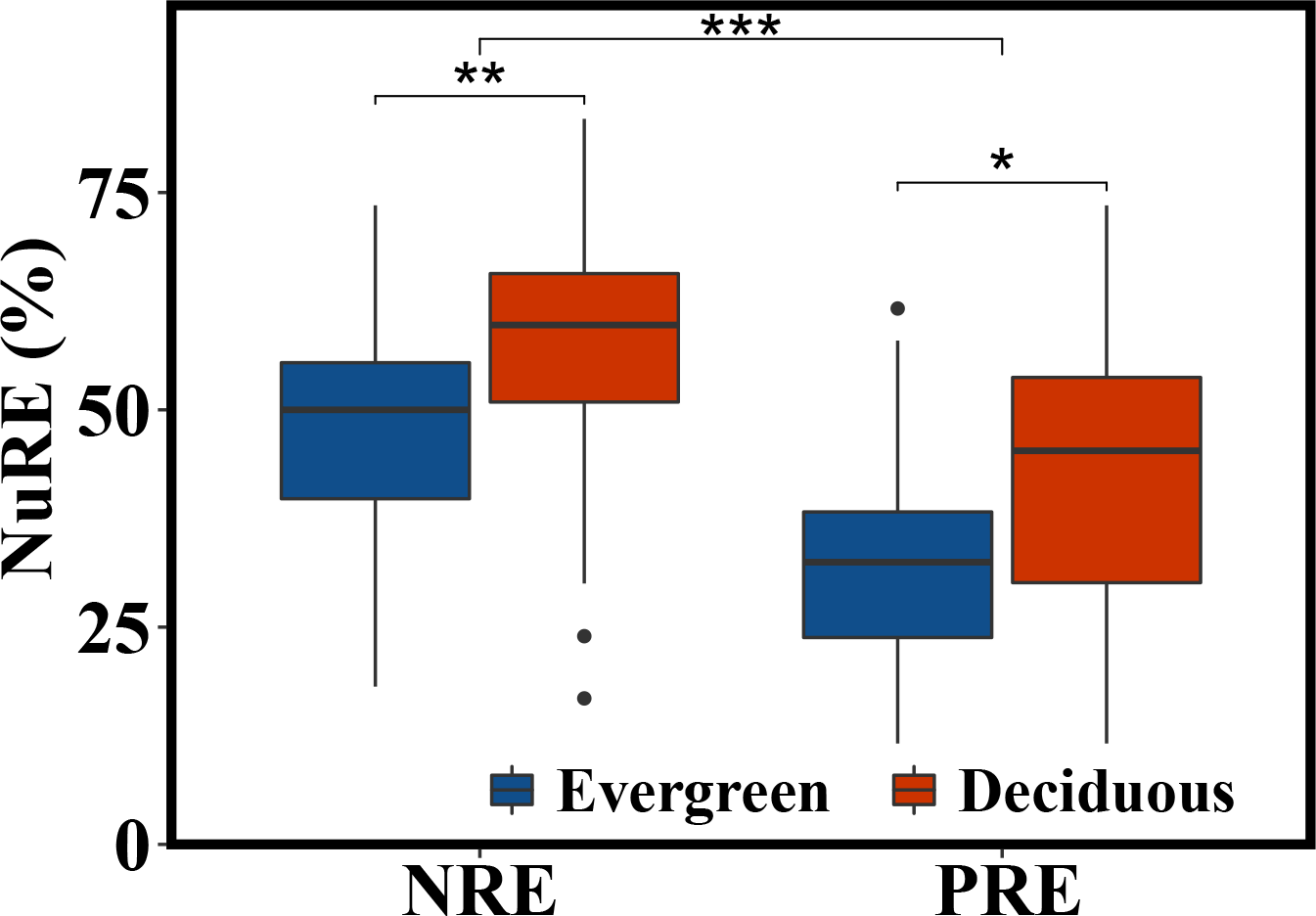
NRE and PRE of evergreen and deciduous trees. *p < 0.05, **p < 0.01, ***p < 0.001.

**Figure 3:**
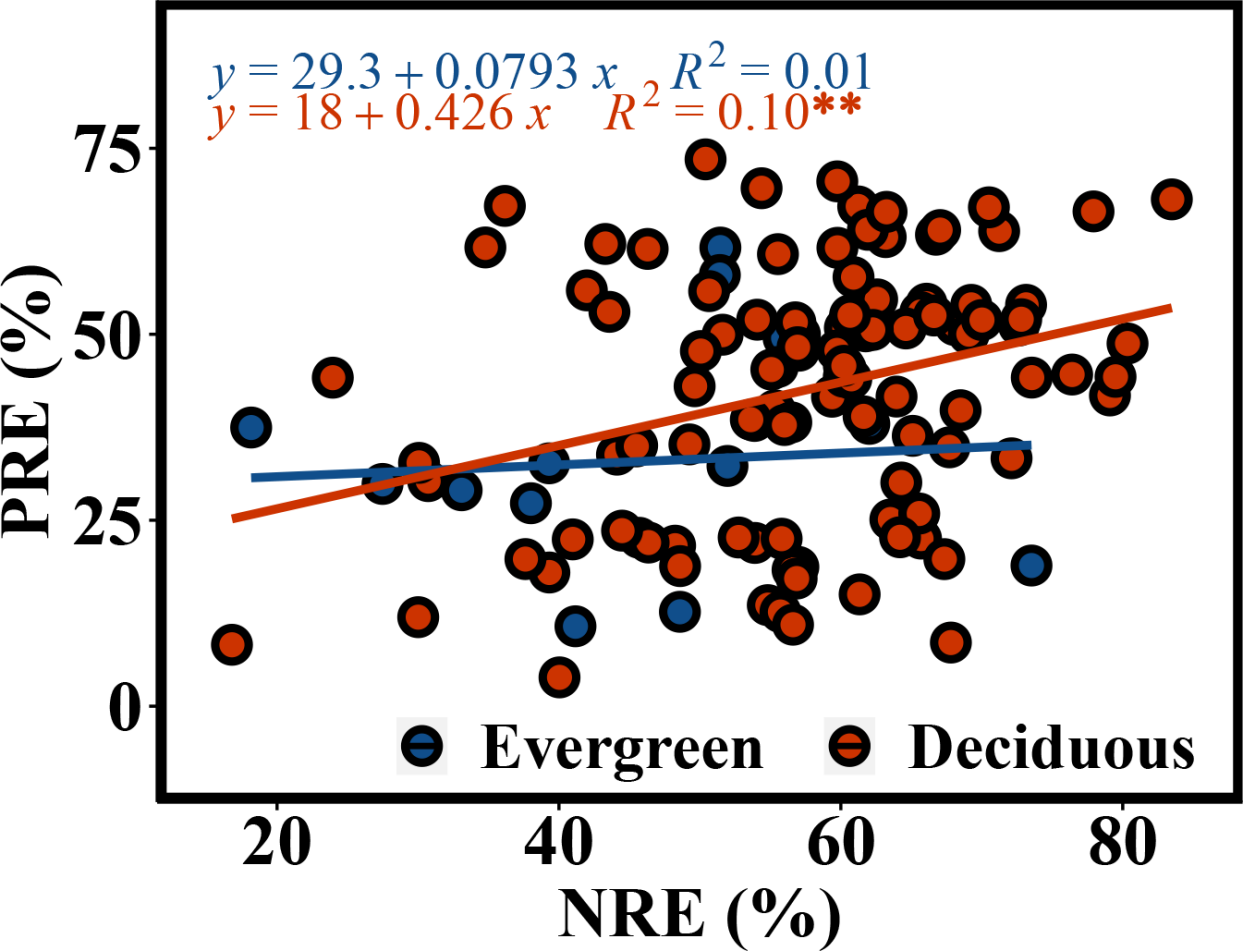
Correlated relationship between NRE and PRE of evergreen and deciduous trees.

The NRE and PRE of the evergreen and deciduous trees were 56.1 ± 13.1% and 41.1 ± 17.0%, respectively (Fig. 2). The PRE was significantly lower than the NRE (*F* = 58.733, *p* = 1.17*e* − 11). The NRE (*F* = 10.114, *p* = 0.002) and PRE (*F* = 4.991, *p* = 0.015) were significantly different between the evergreen and deciduous trees, and those of the evergreen trees were significantly lower than those of the deciduous trees (Fig. 2). The NRE was significantly positively correlated with the PRE among the deciduous trees (*F* = 10.68, *p* = 0.002) (Fig. 3). However, the NRE had no obvious relationship with the PRE among the evergreen trees.

### Soil nutrient concentrations

According to the results, there were no significant differences in the soil nutrients between the evergreen trees and deciduous trees (Table 3).

**Table 3:** Soil nutrient concentrations and N:P of the evergreen and deciduous trees.

**Life form**	**n**	**pH**	**TN** **(g** **kg** ^−1^ **)**	**TP** **(g** **kg** ^−1^ **)**	**TN:TP**	**NH** ${}_{4}^{+}$ **-N** **(mg** **kg** ^−1^ **)**	**NO** ${}_{3}^{-}$ **-N** **(mg** **kg** ^−1^ **)**	**NO** ${}_{2}^{-}$ **-N** **(mg** **kg** ^−1^ **)**	**AP** **(mg** **kg** ^−1^ **)**
Evergreen	18	8.45 ± 0.12	0.63 ± 0.20	0.60 ± 0.15	1.11 ± 0.40	12.45 ± 2.15	14.84 ± 8.25	0.15 ± 0.06	7.39 ± 1.74
Deciduous	102	8.48 ± 0.17	0.59 ± 0.27	0.53 ± 0.16	1.26 ± 0.96	11.50 ± 2.26	13.93 ± 7.80	0.14 ± 0.05	7.19 ± 1.81

**Notes.**

n sampling point number

### Relationship between leaf indexes and soil nutrients in the two life forms

**Figure 4:**
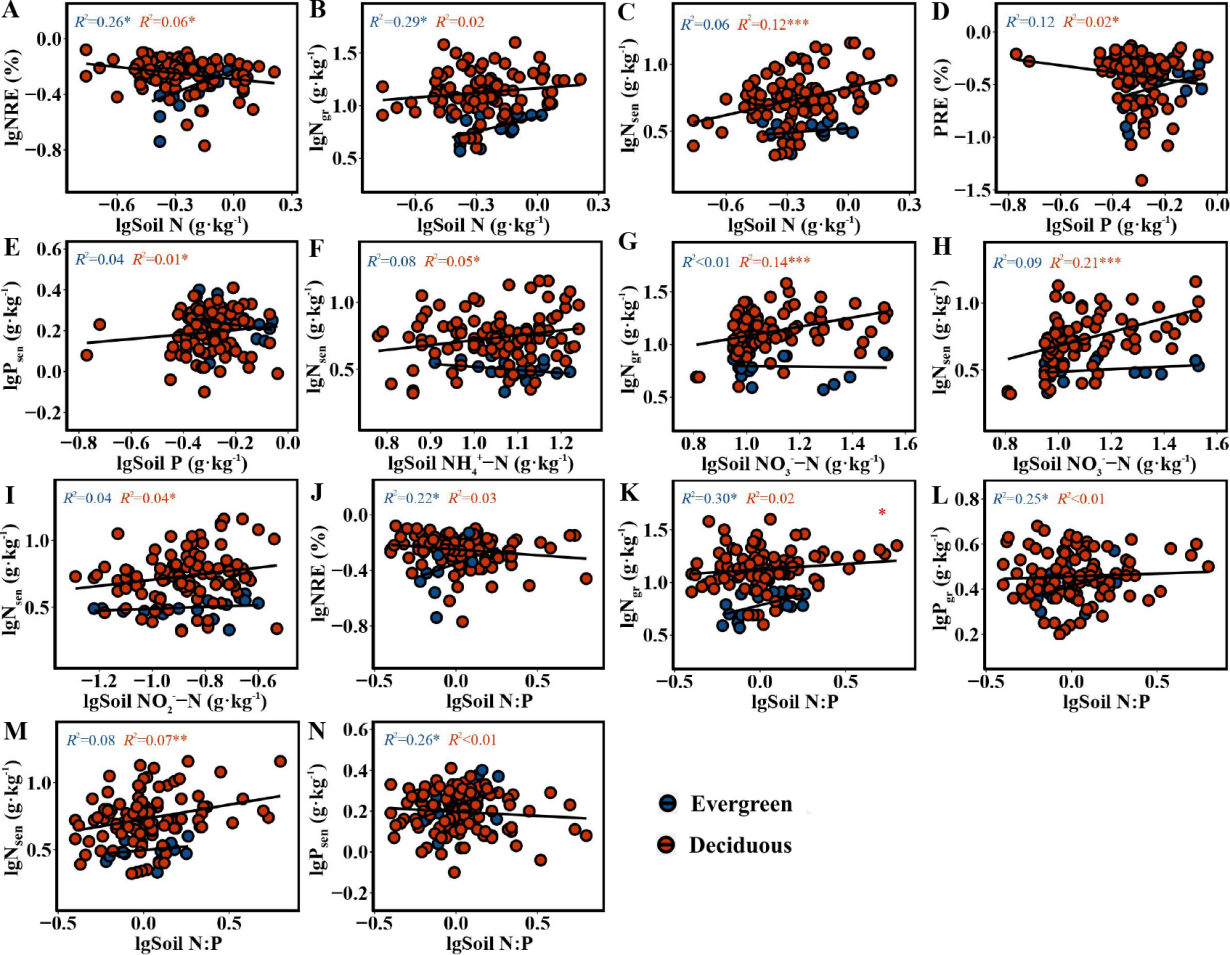
(A–N) The correlation figure of leaf NuRE, N and P concentrations and soil nutrient concentrations and N:P in evergreen and deciduous plants.

The NRE and PRE in the two life forms were distinctly correlated with soil nutrient concentrations and N:P. For the evergreen trees, the NRE (*F* = 5.628, *p* = 0.031) and N_gr_ (*F* = 6.393, *p* = 0.022) had a positive relationship with soil N (Figs. 4A–4B). The NRE (*F* = 4.529, *p* = 0.049), N_gr_(*F* = 6.831, *p* = 0.019), P_gr_ (*F* = 5.231, *p* = 0.036) and P_sen_ (*F* = 5.673, *p* = 0.030) had a significantly positive relationship with soil N:P (Figs. 4J–4L and 4N). For deciduous trees, the NRE had a greatly negative relationship with soil N (*F* = 6.307, *p* = 0.014) (Fig. 4A), and the PRE had a similar relationship with soil P (*F* = 5.905, *p* = 0.017) (Fig. 4D). N_sen_ had a positive relationship with soil N, NH${}_{4}^{+}$-N, NO${}_{3}^{-}$-N, NO${}_{2}^{-}$-N and N:P (*F* = 14.05, *p* = 0.0003; *F* = 5.151, *p* = 0.025; *F* = 43.76, *p* = 1.86*e* − 09; *F* = 4.116, *p* = 0.045; *F* = 8.075, *p* = 0.005) (Figs. 4C, 4F, 4H, 4I and 4M). N_gr_ had a distinctly positive relationship with soil NO${}_{3}^{-}$-N (*F* = 26.87, *p* = 1.14*e* − 06) (Fig. 4G). P_sen_ had a significantly positive relationship with soil P (*F* = 5.186, *p* = 0.025) (Fig. 4E).

**Figure 5:**
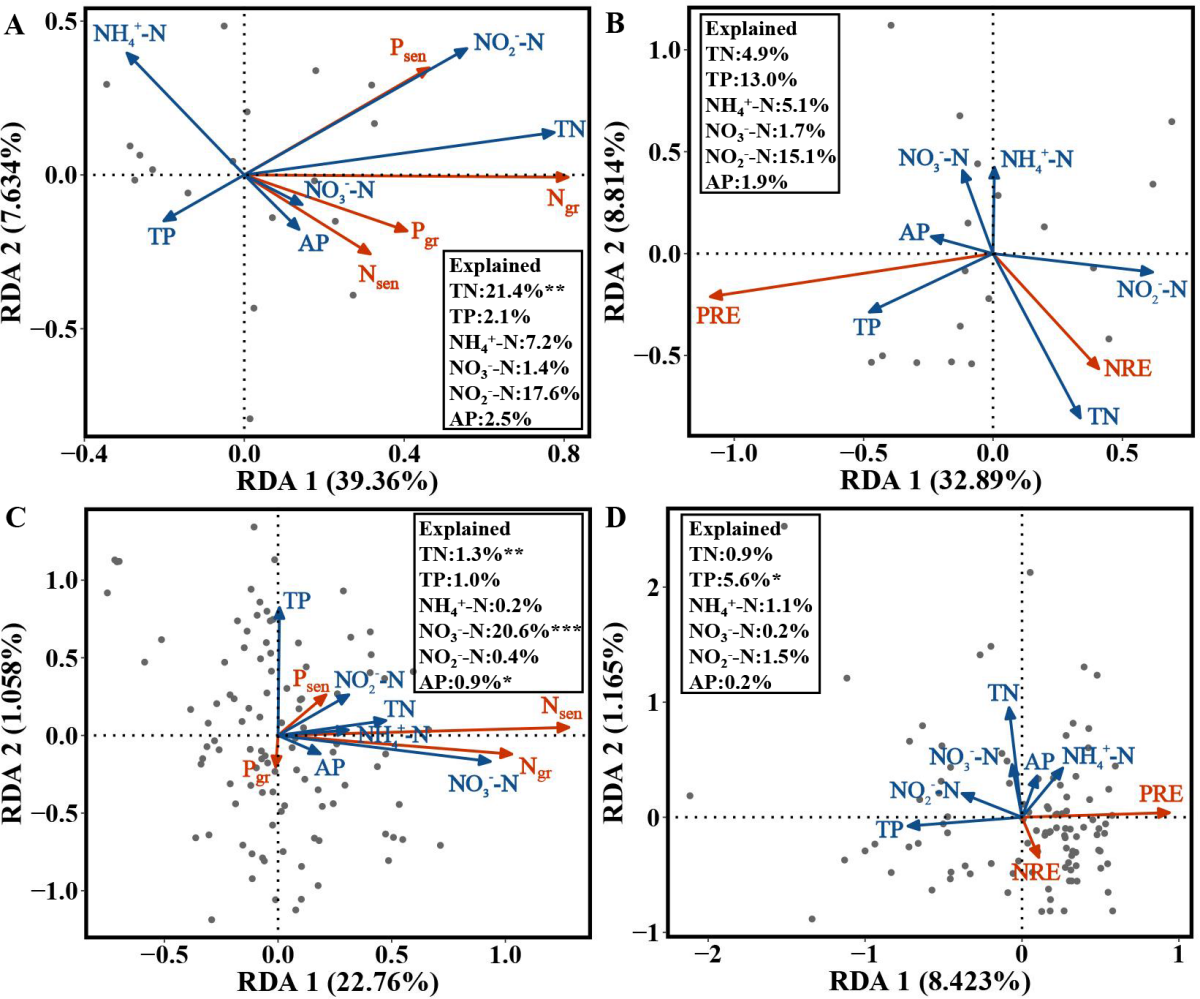
RDA of the NuRE, leaf N and P concentrations and soil nutrient concentrations in evergreen and deciduous plants. (A) N_gr_, P_gr_, N_sen_, P_sen_ and soil nutrient concentrations in evergreen trees. (B) NuRE and soil nutrient concentrations in evergreen trees. (C) N_gr_, P_gr_, N_sen_, P_sen_ and soil nutrient concentrations in deciduous trees. (D) NuRE and soil nutrient concentrations in deciduous trees.

For the evergreen trees, the amount of variation in N_gr_, P_gr_, N_sen_ and P_sen_ in the first and second axes explained by the RDA was 39.36% and 7.634%, respectively. The cumulative amount of variation explained by the RDA was 46.994% (Fig. 5A). N_gr_, P_gr_, N_sen_ and P_sen_ were significantly correlated with soil TN (*F* = 5.5482, *p* = 0.002), and the RDA explained 21.4% of their variation (Fig. 5A). The variation in NRE and PRE explained by the first and second axes of the RDA was 32.89% and 8.814%, respectively. The cumulative amount of variation was 41.704% (Fig. 5B).

For the deciduous trees, the first and second axes of the RDA explained 22.76% and 1.058% of the variation in N_gr_, P_gr_, N_sen_ and P_sen_, respectively. The cumulative amount of variation was 23.818% (Fig. 5C). N_gr_, P_gr_, N_sen_ and P_sen_ were substantially associated with soil TN (*F* = 6.9873, *p* = 0.004), NO${}_{3}^{-}$-N(*F* = 17.8502, *p* = 0.001) and AP (*F* = 3.0538, *p* = 0.040), and the RDA explained 1.3%, 20.6% and 0.09% of their variation, respectively (Fig. 5C). The first and second axes of RDA explained 8.423% and 1.165% of the variation in the NRE and PRE, respectively. The cumulative amount of variation was 9.588% (Fig. 5D). The PRE and NRE were distinctly related to the soil TP ( *F* = 4.7381, *p* = 0.025), and their variation was 5.6% (Fig. 5D). All the data accurately reflected the relationship between leaf N_gr_, P_gr_, N_sen_, P_sen_, NuRE and soil nutrients.

## Discussion

### N and P concentrations and N:P in leaves

Leaf nutrient concentration is a key indicator of plant nutritional status (Chen et al., 2021a; Chen et al., 2021b). In our research, the green leaf N concentration (13.38 g kg^−1^) was lower than the average N concentration (17.57 g kg^−1^) in woody plants in the north-south transect of eastern China (NSTEC) (Ren et al., 2007). Plants mainly obtain P and N from the soil (Zhu et al., 2016; Zhang et al., 2018), and the leaves in urban green spaces are removed regularly every year, resulting in serious soil N loss (Hu et al., 2022). The soil N concentration in this area was low (TN = 0.61 g kg^−1^), so the leaves obtained less N from the soil. Since plants in southern China are generally limited by P and the soil P concentration in northern China is relatively sufficient, the leaf P concentration (2.90 g kg^−1^) in this area was relatively higher than that in woody plants (1.39 g kg^−1^) in the NSTEC (Ren et al., 2007).

In this research, the green leaf P and N concentrations in the deciduous trees were significantly higher than those in the evergreen trees. This result might be attributed to the fact that deciduous trees need to accumulate more N and P in a short time to complete growth-related activities. Due to the longer leaf life cycle of evergreen trees, they take much more time to conserve nutrients (Yan, Zhu & Yang, 2018). Compared with evergreen trees, deciduous trees had higher levels of litter decomposition and nutrient absorption (Wu et al., 2012). The calculated Nu_sen_ was also considered the nutrient utilization efficiency (Tang et al., 2013). In this research, the senesced leaf N concentration in the evergreen trees was significantly lower than that in the deciduous trees, indicating that the N utilization efficiency was higher than the P utilization efficiency (Tang et al., 2013) in this area. They were consistent with hypothesis 1.

In most terrestrial ecosystems, the availability of N and P limits the growth of plants. The concentrations of N and P in green leaves and the N:P can be used as a standard of plant nutritional limitation (Tian, Yan & Fang, 2021). Plants are limited by N when N:P <14 and when the N concentration is <20.0 mg g^−1^ and the P concentration is >1 mg g^−1^ (Wu et al., 2012). In this study, the leaf N:P in the two life forms was less than 14. Additionally, the leaf N concentrations were less than 20.0 mg g^−1^ in most trees, and the P concentrations were more than 1 mg g^−1^ in 40 trees. The N concentrations were lower than those of the same tree species in other areas (Wang et al., 2013), and the N:P was lower than that of 753 species in China (Dong et al., 2023). Combined with the field investigation, according to the growth and physiological characteristics of the trees, we had reason to believe that the garden trees in this area were limited by N. In addition, the N:P in the deciduous trees was higher than that in the evergreen trees, indicating that the evergreen trees were more sensitive to being limited by N than the deciduous trees. The results also showed that under the same urban garden background, N limitation was different between the evergreen and deciduous trees and which was consistent with hypothesis 2. The reason for the difference in N limitation between the two life forms in the same area may be the genetic characteristics of the tree species themselves or the variation in suitable survival to the external environment formed in the long-term evolution process.

### Differences in NRE and PRE in the two life forms

Senesced leaves fall to the ground and gradually breakdown into inorganic nutrients due to physical and microbiological processes, and these remineralized nutrients will eventually be absorbed by plants(biogeochemical cycle) (Han et al., 2013). The NRE (56.13%) was higher than the PRE (41.14%), indicating that plant absorption of N was higher than that of P. However, these NRE and PRE values are lower than those (62.1% and 64.9%, respectively) of global terrestrial forest ecosystems. This result might be due to the differences in the trees or habitats selected for this research and how urbanization associated with N and P inputs has altered the soil N and P status and plant N and P absorption (Hu et al., 2011). Additionally, when plants are restricted by N, they tend to accomplish total N absorption but inadequate P absorption (Chen & Chen, 2021).

Compared with the evergreen trees, the deciduous trees had much higher NRE and PRE, indicating that nutrient utilization in the evergreen trees was lower than that in the deciduous trees (Chen & Chen, 2021). P resorption variation is more affected by climate and soils, while N resorption variance relates more to plant functional type (Tang et al., 2013). Evergreen trees, a type of highly specialized plant, have developed an adaptation technique to thrive in nutrient-poor soil (Reich, Walters & Ellsworth, 1992). In barren soil, extending the leaf lifespans of evergreen trees can allow the growth rate to reach its maximum (Reich, Walters & Ellsworth, 1997) and nutrient losses to reach their minimum during leaf senescence and shedding (Aerts, 1995). During the growth season, deciduous trees produce more leaves, and they shed their leaves annually (Reich et al., 1997). Thus, deciduous trees often invest more nutrients to support rapid growth during shorter growing seasons (Brant & Chen, 2015). Thus, in this study, in comparison to evergreen trees, deciduous trees had much higher NRE and PRE. These findings indicated that the two life-form garden tree species have different N and P utilization strategies, which is consistent with hypothesis 1. Additionally, the variation in the decomposability-related characteristics of the litter (such as lignin: N and N:P) and the habitat climate (temperature/precipitation) may work in conjunction to affect the availability of soil nutrients and, consequently, the levels of NRE and PRE in evergreen and deciduous trees (McGroddy, Daufresne & Hedin, 2004).

In addition, in the deciduous trees, the NRE was positively associated with the PRE, indicating that N absorption increases concurrently with P absorption. This result was a crucial premise for stoichiometry control (Chen & Chen, 2021), further showing that the deciduous trees had a synergistic effect on N and P. A meta-analysis by Aerts(Aerts, 1995) also showed that NRE and PRE have a strong association with one another. Our result is consistent with this finding.

### Relationship between leaf N_**gr**_, P_**gr**_, N_**sen**_, P_**sen**_, NuRE and soil nutrients in the two life forms

Soil nutrient availability and leaf nutrient status are considered important control factors for nutrient absorption processes (Xu et al., 2020; Chen & Chen, 2021). Our findings demonstrated that soil nutrients had a considerable impact on leaf N and P concentrations as well as on NuRE in the two life forms. The availability of N and P had a significant impact on how soil nutrients were utilized, which had an impact on NuRE (Liu et al., 2020).

For the evergreen trees, the NRE and N_gr_ were positively correlated with soil TN, indicating that the evergreen trees did not reduce their resorption levels to adapt to the increase in soil N concentration. The increase in the NRE was mainly due to the decrease in the N_gr_. We predicted that the evergreen trees would store a large amount of N in their green leaves when the soil N supply was sufficient or possibly N-limited, while the N in senesced leaves was almost unaffected, thus improving the NRE. Soil N:P can be used as a measure of N saturation, showing the availability of nutrients for plant growth (Stark, Ylanne & Tolvanen, 2018). The P_gr_ and P_sen_ were positively related to soil N:P, indicating that the evergreen trees adopted a nutrient utilization strategy with high concentration and high return to maintain their normal physiological activities when soil P was relatively scarce compared with N. The NRE and N_gr_ in the evergreen trees were positively associated with soil N:P, and the RDA (Fig. 5A) showed that soil TN had the highest variation, which indicated that soil TN supply was the key factor affecting the N and P concentrations in the evergreen tree leaves.

For the deciduous trees, the NRE was negatively correlated with soil TN, while the PRE was negatively associated with soil TP, indicating that the NRE and PRE decreased with increasing soil TN and TP. We concluded that the deciduous trees did not need higher resorption efficiency to maintain N and P concentrations when the N and P supply in the soil was sufficient. The result is consistent with that of Kobe, Lepczyk & Iyer (2005). N_sen_ was positively associated with soil N in various forms and N:P, indicating that N_sen_ increased with increasing soil N concentration and availability. The deciduous trees mainly affected the N_sen_ through the soil N concentration, which further affected the NRE. The dominant soil factors affecting the N and P concentrations in the deciduous trees were soil TN and NO${}_{3}^{-}$-N (Fig. 5C), and soil TP was the main soil factor affecting the NuRE (Fig. 5D), indicating that soil N mainly influenced the leaf N and P concentrations; however, soil P mainly affected the leaf NuRE in the deciduous trees. The evergreen trees experienced more variation in soil accumulation than the deciduous trees, demonstrating that evergreen trees were more sensitive to soil N and P concentrations than deciduous trees, which is consistent with hypothesis 3.

## Conclusions

This research presents an advanced understanding of the N and P nutrient utilization strategies of two life-form tree species and their responses to soil nutrients in urban ecosystems. The N concentrations of green and senesced leaves were much higher in the deciduous trees than in the evergreen trees. The P concentration of green leaves was much higher in the deciduous trees than in the evergreen trees. The two life-form tree species were both N limited, and evergreen trees were more sensitive to N limitation than deciduous trees. The NRE and PRE of the deciduous trees were significantly higher than those of the evergreen trees. The NRE of the deciduous trees was significantly positively correlated with the PRE. As the soil N and P concentrations increased, the NuRE of the evergreen trees increased, but that of the deciduous trees decreased. Soil TN mainly influenced the leaf N and P concentrations in evergreen trees. Soil TN and NO${}_{3}^{-}$-N mainly influenced the leaf N and P concentrations, while soil TP mainly influenced the NuRE in deciduous trees. Compared with the deciduous trees, the evergreen trees were more sensitive to the feedback of soil N and P concentrations. Our findings fill a gap in the understanding of woody plant nutrient resorption in urban ecosystems and provide a theoretical basis for the selection of garden tree species to some extent. Furthermore, these findings can serve as the foundation for the construction of energy-saving ecological garden cities and can be used to incorporate these nutrient absorption processes into future biogeochemical models.

##  Supplemental Information

10.7717/peerj.15738/supp-1 Table S1 N and P concentriations and NRE and PRE of different tree species

10.7717/peerj.15738/supp-2 Table S2 Soil nutrient concentrations of different tree species

10.7717/peerj.15738/supp-3 Supplemental Information 3 Data and code

## Additional Information and Declarations

### Data Availability

The following information was supplied regarding data availability: The raw data and code are in the Supplemental Files.
