# Supplementary material for: Leaf nutrient resorption of two life-form tree species in urban gardens and their response to soil nutrient availability
Source: PeerJ. 2023 Jul 19;11:e15738. doi: 10.7717/peerj.15738 (PMC10362843; doi:10.7717/peerj.15738)
Supplement: Table S1 [file peerj-11-15738-s001.docx]

**Appendix Table S1 N and P concentriations and NRE and PRE of different tree species.**

| **Life form** | **Tree species** | **N_gr_**  **(g·kg^-1^)** | **P_gr_**  **(g·kg^-1^)** | **N_sen_**  **(g·kg^-1^)** | **P_sen_**  **(g·kg^-1^)** | **NRE(%)** | **PRE(%)** |
| --- | --- | --- | --- | --- | --- | --- | --- |
| Evergreen | *P. tabulaeformis* | 5.00±1.83 | 2.55±0.22 | 2.84±0.27 | 1.54±0.25 | 41.66+10.86 | 31.56+5.95 |
|  | *P. bungeana* | 4.26±1.47 | 2.82±0.30 | 3.01±0.08 | 1.69±0.13 | 28.32+10.59 | 33.33+3.84 |
|  | *P. meyeri* | 5.84±0.65 | 3.01±0.56 | 3.07±0.23 | 1.73±0.27 | 47.57+3.37 | 39.78+19.94 |
|  | *S. chinensis* | 7.25±0.87 | 4.26±0.10 | 3.66±0.29 | 2.59±0.72 | 48.91+7.91 | 14.05+4.16 |
|  | *S. procumbens* | 8.22±0.21 | 2.97±0.21 | 3.36±0.28 | 1.68±0.10 | 59.13+3.05 | 43.07+5.87 |
|  | *E. kiautschovicus* | 7.68±0.42 | 2.44±0.31 | 3.13±0.77 | 1.52±0.38 | 58.97+12.61 | 36.39+19.85 |
| Deciduous | *S. japonica* | 20.44±3.81 | 3.11±0.03 | 11.13±1.87 | 2.12±0.05 | 45.19+3.77 | 22.00+0.42 |
|  | *R. pseudoacacia* | 23.24±4.51 | 4.19±0.20 | 10.29±0.88 | 1.97±0.08 | 53.64+14.20 | 24.91+8.80 |
|  | *E. maackii* | 11.32±0.89 | 3.76±0.09 | 4.79±0.53 | 2.11±0.23 | 57.61+5.46 | 22.03+3.43 |
|  | *P. tomentosa* | 14.34±1.77 | 2.26±0.13 | 5.25±0.13 | 1.20±0.02 | 62.92+5.10 | 51.63+3.37 |
|  | *P. alba* | 12.49±1.91 | 2.52±0.12 | 5.36±0.50 | 1.72±0.22 | 56.05+10.32 | 31.76+9.12 |
|  | *G. biloba* | 5.31±1.41 | 3.59±0.87 | 2.45±0.16 | 1.97±0.47 | 52.45+8.78 | 40.91+8.24 |
|  | *M. micromalus* | 12.73±0.82 | 2.36±0.67 | 5.14±0.24 | 1.28±0.07 | 59.54+2.17 | 56.01+9.69 |
|  | *P. cerasifera* | 9.50±1.41 | 2.49±0.46 | 3.50±0.32 | 1.47±0.12 | 62.35+8.60 | 55.41+7.37 |
|  | *F. chinensis* | 16.47±3.79 | 2.09±0.51 | 5.13±0.85 | 1.49±0.17 | 68.30+4.62 | 34.71+23.48 |
|  | *S. babylonica* | 15.50±3.14 | 1.86±1.77 | 6.41±1.19 | 1.05±0.12 | 57.30+13.39 | 60.78+4.23 |
|  | *S. matsudana* | 14.24±0.05 | 2.89±0.07 | 6.28±0.29 | 1.90±0.35 | 55.87+0.58 | 33.48+9.53 |
|  | *A. julibrissin* | 28.65±12.48 | 3.02±0.79 | 13.09±1.19 | 1.77±0.16 | 49.36+18.69 | 38.90+10.97 |
|  | *A. negundo* | 31.28±7.86 | 2.98±0.02 | 8.56±1.17 | 1.85±0.20 | 71.11+10.08 | 48.42+2.94 |
|  | *A. truncatum* | 11.58±1.68 | 2.49±0.27 | 4.05±0.90 | 1.36±0.18 | 64.73+8.01 | 51.47+0.63 |
|  | *C. coggygria* | 9.48±0.34 | 3.07±0.66 | 3.01±0.16 | 1.36±0.26 | 68.26+1.12 | 52.59+12.17 |
|  | *K. paniculata* | 10.25±1.59 | 3.56±0.18 | 5.27±0.30 | 2.06±0.23 | 47.69+9.65 | 18.59+1.33 |
|  | *M. alba* | 17.67±0.67 | 2.52±1.54 | 7.36±0.65 | 1.89±0.10 | 58.34+3.73 | 49.52+4.68 |
|  | *B. papyrifera* | 23.62±4.83 | 3.36±0.42 | 9.84±1.68 | 2.12±0.48 | 57.93+3.76 | 49.72+11.51 |
|  | *E. ulmoides* | 14.07±0.06 | 3.42±0.37 | 5.32±0.96 | 1.83±0.33 | 62.22+7.50 | 26.94+10.12 |
|  | *R. typhina* | 18.91±2.21 | 3.24±1.06 | 4.82±0.89 | 1.50±0.13 | 74.04+7.76 | 48.21+16.09 |
|  | *Q. wutaishanica* | 12.03±2.02 | 2.80±0.71 | 5.44±0.90 | 1.78±0.44 | 52.30+19.42 | 17.63+7.32 |
|  | *C. pinnatifida* | 10.52±0.06 | 2.53±0.00 | 5.29±0.65 | 1.41±0.64 | 49.67+5.95 | 54.40+8.53 |
|  | *A. sibirica* | 11.00±2.82 | 2.52±0.40 | 5.38±0.94 | 1.70±0.23 | 48.30+21.09 | 44.15+0.14 |
|  | *A. davidiana* | 11.83±3.90 | 2.71±1.18 | 5.57±0.86 | 1.97±0.26 | 47.22+26.40 | 27.84+22.59 |
|  | *C. serrulata* | 12.30±1.27 | 3.60±0.05 | 2.95±0.49 | 1.81±0.18 | 76.00+3.71 | 40.73+6.42 |
|  | *A. triloba* | 10.87±2.86 | 2.30±0.37 | 4.81±1.03 | 1.24±0.27 | 54.24+13.25 | 50.26+2.64 |
|  | *S. oblata* | 11.75±0.38 | 1.67±0.68 | 5.92±1.28 | 0.97±0.19 | 49.49+12.90 | 68.27+4.82 |
|  | *S. japonica var. japonica f. pendula* | 20.47±1.97 | 3.03±0.43 | 8.72±4.50 | 1.41±0.34 | 58.43+20.49 | 60.22+7.68 |
|  | *F. suspensa* | 12.26±3.78 | 2.37±0.05 | 5.65±0.40 | 1.28±0.28 | 50.23+17.47 | 46.08+12.58 |
|  | *H. syriacus* | 15.60±0.87 | 2.71±1.02 | 7.09±1.76 | 1.74±0.23 | 54.50+12.61 | 38.70+31.89 |
|  | *S. sorbifolia* | 20.93±3.21 | 3.15±0.02 | 6.81±0.65 | 1.70±0.28 | 66.64+6.89 | 52.77+8.73 |
|  | *L. japonica* | 8.81±0.47 | 2.65±0.63 | 4.65±0.10 | 1.25±0.13 | 47.14+2.19 | 46.47+13.61 |
|  | *C. alba* | 12.57±1.86 | 3.10±0.12 | 4.07±1.24 | 1.28±0.13 | 65.87+15.49 | 69.45+1.22 |
|  | *B. thunbergii* | 4.86±0.10 | 3.05±0.94 | 2.15±0.08 | 1.70±0.11 | 55.71+0.89 | 12.40+1.33 |
