# Supplementary material for: Leaf nutrient resorption of two life-form tree species in urban gardens and their response to soil nutrient availability
Source: PeerJ. 2023 Jul 19;11:e15738. doi: 10.7717/peerj.15738 (PMC10362843; doi:10.7717/peerj.15738)
Supplement: Table S2 [file peerj-11-15738-s002.docx]

**Appendix Table S2 Soil nutrient concentrations of different tree species.**

| **Life form** | **Tree species** | **pH** | **TN**  **(g·kg^-1^)** | **TP**  **(g·kg^-1^)** | **NH_4_^+^-N**  **(mg·kg^-1^)** | **NO_3_^-^-N**  **(mg·kg^-1^)** | **NO_2_^-^-N**  **(mg·kg^-1^)** | **AP**  **(mg·kg^-1^)** |
| --- | --- | --- | --- | --- | --- | --- | --- | --- |
| Evergreen | *P. tabulaeformis* | 8.61±0.11 | 0.51±0.02 | 0.81±0.12 | 13.23±0.89 | 9.79±0.58 | 0.11±0.05 | 5.97±0.43 |
|  | *P. bungeana* | 8.51±0.06 | 0.42±0.01 | 0.55±0.03 | 14.43±2.64 | 21.77±2.25 | 0.12±0.02 | 9.32±2.25 |
|  | *P. meyeri* | 8.40±0.48 | 0.75±0.01 | 0.50±0.07 | 12.26±0.38 | 9.70±0.44 | 0.08±0.03 | 7.00±1.83 |
|  | *S. chinensis* | 8.27±0.35 | 0.74±0.09 | 0.48±0.07 | 12.42±1.54 | 19.53±10.96 | 0.21±0.04 | 7.54±0.78 |
|  | *S. procumbens* | 8.49±0.21 | 0.93±0.14 | 0.78±0.09 | 12.61±1.90 | 17.57±11.73 | 0.17±0.04 | 8.48±0.49 |
|  | *E. kiautschovicus* | 8.41±0.51 | 0.45±0.08 | 0.47±0.03 | 9.77±1.57 | 10.69±2.45 | 0.17±0.05 | 6.01±0.48 |
| Deciduous | *S. japonica* | 8.57±0.21 | 0.70±0.07 | 0.56±0.15 | 13.20±3.18 | 18.78±11.38 | 0.20±0.07 | 7.20±1.11 |
|  | *R. pseudoacacia* | 8.30±0.25 | 0.52±0.07 | 0.52±0.05 | 15.80±0.82 | 12.56±1.46 | 0.14±0.01 | 6.68±0.68 |
|  | *E. maackii* | 8.35±0.33 | 0.82±0.39 | 1.11±0.07 | 11.12±0.63 | 10.35±1.04 | 0.07±0.01 | 6.23±1.29 |
|  | *P. tomentosa* | 8.47±0.22 | 0.59±0.02 | 0.38±0.03 | 9.07±2.01 | 10.19±0.23 | 0.08±0.03 | 7.04±1.03 |
|  | *P. alba* | 8.33±0.27 | 0.46±0.06 | 0.45±0.09 | 13.87±1.53 | 18.01±7.63 | 0.19±0.04 | 7.38±1.80 |
|  | *G. biloba* | 8.64±0.14 | 0.50±0.03 | 0.43±0.08 | 13.15±0.90 | 11.84±2.06 | 0.14±0.04 | 8.37±2.87 |
|  | *M. micromalus* | 8.63±0.10 | 0.48±0.09 | 0.51±0.10 | 11.38±1.20 | 9.80±0.22 | 0.12±0.01 | 6.05±0.55 |
|  | *P. cerasifera* | 8.65±0.06 | 0.21±0.03 | 0.43±0.03 | 11.21±3.01 | 9.36±0.16 | 0.10±0.01 | 5.35±0.10 |
|  | *F. chinensis* | 8.56±0.08 | 0.53±0.07 | 0.52±0.07 | 10.77±3.62 | 15.19±6.28 | 0.18±0.03 | 7.80±0.83 |
|  | *S. babylonica* | 8.13±0.45 | 1.12±0.07 | 0.49±0.15 | 14.02±1.11 | 18.38±8.59 | 0.16±0.05 | 6.64±0.54 |
|  | *S. matsudana* | 8.51±0.01 | 0.34±0.01 | 0.54±0.12 | 15.16±1.86 | 9.89±0.10 | 0.17±0.03 | 5.21±0.16 |
|  | *A. julibrissin* | 8.01±0.11 | 1.02±0.27 | 0.56±0.13 | 15.00±1.57 | 44.07±4.02 | 0.21±0.04 | 12.18±1.35 |
|  | *A. negundo* | 8.28±0.27 | 0.39±0.05 | 0.60±0.17 | 7.86±0.38 | 14.56±0.44 | 0.13±0.02 | 5.33±0.22 |
|  | *A. truncatum* | 8.31±0.50 | 0.51±0.02 | 0.49±0.09 | 13.88±1.97 | 10.57±0.88 | 0.14±0.05 | 6.77±0.73 |
|  | *C. coggygria* | 8.60±0.01 | 0.46±0.25 | 0.71±0.24 | 12.45±0.82 | 9.12±0.26 | 0.09±0.02 | 5.23±0.58 |
|  | *K. paniculata* | 8.65±0.03 | 0.33±0.08 | 0.55±0.08 | 10.12±2.66 | 9.86±0.71 | 0.10±0.03 | 5.24±0.25 |
|  | *M. alba* | 8.49±0.26 | 1.34±0.31 | 0.51±0.10 | 12.74±3.26 | 23.12±7.55 | 0.15±0.00 | 9.48±1.99 |
|  | *B. papyrifera* | 8.44±0.22 | 0.57±0.04 | 0.62±0.28 | 9.66±1.69 | 12.96±4.71 | 0.10±0.02 | 7.08±1.14 |
|  | *E. ulmoides* | 8.55±0.05 | 0.68±0.08 | 0.50±0.05 | 10.00±2.64 | 15.52±3.41 | 0.10±0.02 | 5.75±0.70 |
|  | *R. typhina* | 8.60±0.03 | 0.34±0.02 | 0.54±0.05 | 14.88±1.77 | 9.85±0.84 | 0.14±0.05 | 5.90±0.48 |
|  | *Q. wutaishanica* | 8.40±0.21 | 0.49±0.17 | 0.57±0.10 | 8.90±1.21 | 13.60±3.61 | 0.24±0.08 | 6.18±0.45 |
|  | *C. pinnatifida* | 8.64±0.10 | 1.12±0.02 | 0.53±0.02 | 10.54±0.36 | 10.81±0.42 | 0.18±0.03 | 9.44±0.54 |
|  | *A. sibirica* | 8.59±0.07 | 0.57±0.06 | 0.57±0.07 | 11.74±1.78 | 12.49±2.11 | 0.12±0.03 | 8.16±0.78 |
|  | *A. davidiana* | 8.44±0.02 | 0.60±0.12 | 0.65±0.05 | 9.75±0.82 | 9.64±0.43 | 0.10±0.04 | 7.58±1.34 |
|  | *C. serrulata* | 8.50±0.01 | 0.57±0.00 | 0.44±0.02 | 9.14±0.21 | 13.77±0.30 | 0.14±0.03 | 8.03±0.35 |
|  | *A. triloba* | 8.68±0.13 | 0.36±0.02 | 0.48±0.08 | 10.35±1.70 | 12.48±4.57 | 0.13±0.02 | 6.99±0.44 |
|  | *S. oblata* | 8.65±0.10 | 0.50±0.04 | 0.48±0.01 | 10.61±2.02 | 10.66±1.61 | 0.10±0.02 | 6.47±0.65 |
|  | *S. japonica var. japonica f. pendula* | 8.19±0.55 | 0.94±0.15 | 0.17±0.01 | 12.37±1.37 | 17.94±11.18 | 0.19±0.05 | 8.94±0.37 |
|  | *F. suspensa* | 8.65±0.11 | 0.68±0.08 | 0.65±0.23 | 12.73±1.37 | 13.11±4.78 | 0.13±0.04 | 7.98±2.60 |
|  | *H. syriacus* | 8.53±0.10 | 0.50±0.14 | 0.48±0.05 | 8.31±1.65 | 14.49±6.93 | 0.19±0.01 | 8.11±0.90 |
|  | *S. sorbifolia* | 8.47±0.31 | 0.42±0.11 | 0.45±0.02 | 10.62±1.14 | 18.35±7.26 | 0.18±0.03 | 6.59±0.97 |
|  | *L. japonica* | 8.51±0.29 | 0.60±0.21 | 0.41±0.11 | 13.00±3.21 | 15.94±8.18 | 0.15±0.03 | 8.05±3.03 |
|  | *C. alba* | 8.69±0.04 | 0.32±0.15 | 0.53±0.16 | 10.50±3.71 | 9.77±1.35 | 0.11±0.02 | 6.98±0.83 |
|  | *B. thunbergii* | 8.41±0.44 | 0.47±0.04 | 0.52±0.00 | 7.24±0.44 | 6.51±4.89 | 0.18±0.08 | 8.14±0.18 |
